# Surgical Modification of the Murine Calvaria Osteolysis Model

**DOI:** 10.1155/2015/802697

**Published:** 2015-12-03

**Authors:** Ali Mohammed Al-quhali, Yu Sun, Xizhuang Bai, Zhe Jin, Guibo Yu

**Affiliations:** ^1^Department of Joint Surgery and Sports Medicine, The First Hospital of China Medical University, 155 Nanjing North Street, Shenyang, Liaoning 110001, China; ^2^Key Laboratory of Diagnostic Radiology and Interventional Treatment, The First Hospital of China Medical University, 155 Nanjing North Street, Shenyang, Liaoning 110001, China

## Abstract

The murine calvaria model has been adopted for evaluation of osteolysis and inflammation induced by polyethylene (PE) or metal wear debris. However, this model suffers from several complications. The purpose of our study is to introduce a surgical modification with lower complication rates, thus providing more accurate results. Forty C57/BL6 mice were divided into two groups, both receiving polyethylene particles. Surgical modifications were performed in group 1, and group 2 underwent traditional surgeries. The incidence of fluid leakage was recorded on the operative day. Curst formation, wound dehiscence, and bone exposure were recorded on day 7. Histological osteolysis was demonstrated by HE staining of tissue slices. Micro-CT was used for quantifying evaluation of osteolysis in two groups. Intraoperative fluid leakage was significantly reduced in group 1. Postoperative crust formation, wound dehiscence, and bone exposure were also significantly decreased in group 1. HE staining results revealed obvious osteolysis in group 1 and more obvious osteolysis in group 2. Bone volume fraction (BVF) was (0.32 ± 0.03) in group 1 compared to group 2 (0.24 ± 0.05). Bone mineral density (BMD) was (1.11 ± 0.03) in group 1 compared to group 2 (1.01 ± 0.02). Surgical modifications provide a reliable way for establishment of the murine calvaria osteolysis model.

## 1. Introduction

Aseptic loosening of joint prosthesis induced by polyethylene (PE) and metal wear debris is a disastrous postoperative complication, leading to implant failure and revision surgery [1–5]. Wear debris from the implant results in bone resorption by activation of inflammatory factors and finally loss of fixation [6–8]. Due to the limitation of the access to human joint tissues and implanted prosthesis in early stages [9], the murine calvaria model introduced by Merkel et al. using polyethylene particles [10] and these early previous studies [11] used the traditional surgical techniques for assessment of polyethylene and titanium particles. This murine calvaria model has been widely adopted for evaluation of osteolysis and inflammation and is thought to be sensitive, cost-effective, and time saving. However, traditional surgery techniques for this animal model suffer from several drawbacks, such as obvious fluid leakage and severe skin infection or inflammation, influencing the accuracy and repeatability of correlated research [12]. As reported in previous publication by other researchers, fluid leakage and skin incision are common in the mouse calvaria osteolysis model but ignored or not mentioned in most cases. Without good control of the quality of surgical procedure, fluid leakage will of course lead to the inaccuracy in dose of agents or particles administered, and thus most particles will be left outside incisions but not on the calvaria bone. Besides, skin irritations alone can lead to inflammations of the underlying bone (as there is no other underlying soft tissue between skin and bone in this area), which act as an important confounding factor for analyzing result which is to demonstrate inflammations that are caused only by debris particles. The purpose of this study is to introduce a surgical modification of the conventional murine calvaria osteolysis model with lower complication rates. The modification to the original technique may be seen to be minor; however, we believe that these changes are of importance and could better be taken into consideration in future research.

## 2. Materials and Methods

### 2.1. Particles Preparation

**Figure 1:**
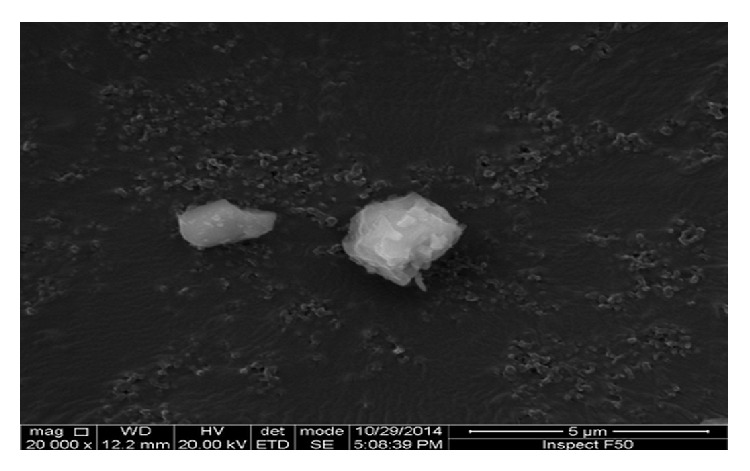
Scanning electron microscopy (SEM) appearance of polyethylene particles (magnification ×20000).

Ultra-high molecular weight polyethylene (UHMWPE) microsize particles as inflammatory agent modeling osteolysis were generously provided by the Institute Of Metal Research (China Academy of Sciences). Particles were evaluated by scanning electron microscope (SEM) as described elsewhere previously [13]. The SEM scanning demonstrated that more than 95% of particles were less than 7 *μ*m in diameter, with a mean size of 3.2 *μ*m (range from <1.0 to 23 *μ*m) (Figure 1). Particles were ultrasonically rinsed in 70% ethanol solution three times. After standard ethylene oxide (EtO) sterilization [14–16], the particles were washed three times in phosphate buffered saline (PBS) and suspended in sterile PBS for injection in concentration of 100 mg/mL. The absence of endotoxin was confirmed using the Limulus Assay Kit (GenScript, (Nanjing) Co.). Finally, the particle suspension was stored at 4°C until use.

### 2.2. Animals

Institutional approval was obtained for all animal procedures from ethical committee of China Medical University. Forty C57/BL6 male mice (provided by Laboratory Animal Center of China Medical University) aged 12–16 weeks with mean weight of 22.7 g (20.3–26.5 g) were housed and fed in our Animal Facility under local vivarium conditions (24°C and 12 h/12 h light/dark cycle) and were housed in quarantine 48 hours prior to the experiment to allow acclimatization. Animals were randomly divided into two groups; each experimental group comprised 20 mice: surgical modifications were performed in group 1, with skin clip assisted posterior incision closure and particle injections adjacent to skin incisions (paraclip technique). Group 2 underwent traditional surgeries, with normal skin suture assisted conventional anterior incision closure and particle injections via skin incision (transsuture technique), served as control.

### 2.3. Surgical Procedure

**Figure 2:**
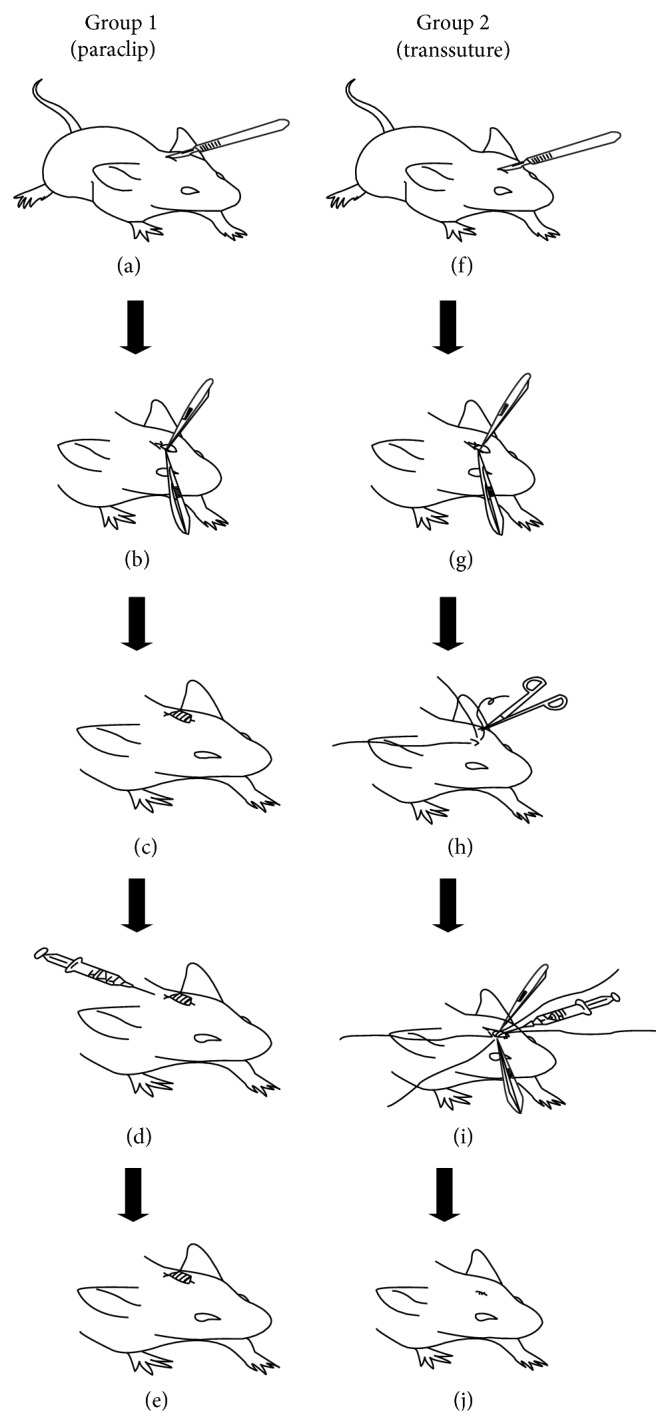
Schematic illustration of surgical process of group 1 (paraclip) and group 2 (transsuture). (a) Posterior skin incision between ears. (b) Periosteum removal. (c) Surgical incision closed with skin clip. (d) Percutaneous injection of UHMWPE particles suspension, 2 mm posterior to the caudal side of incision. (e) Final look of modified model (paraclip). (f) Traditional incision directly above the frontal and parietal bone. (g) Periosteum removal. (h) Interrupted skin sutures without knotting. (i) Direct injection of UHMWPE particles suspension. (j) Final look of traditional model (transsuture) after suture knotting.

Mice were anesthetized by intraperitoneal injection of pentobarbital sodium (50 mg/kg). 1 cm^2^ of mouse head hair was cut by ophthalmic scissor, and then the area was cleaned by normal saline followed by povidone iodine. In group 1, a posterior 3 mm longitudinal skin incision between ears was made; due to good mobility and ductility of skin, the external periosteum of frontal and parietal bone can be easily removed by pulling the incision anteriorly, which provided enough surgical exposure area. Then, the skin incision was closed by 4.5 mm mouse wound clip (SQJ-1, Globalebio. Ltd., Peking, China), followed by percutaneous injection of 100 *μ*L UHMWPE particles suspension to the junction zone of frontal and parietal bone using microsyringe, with the injection sites 2 mm posterior to the caudal side of incision (see Figures 2(a) to 2(e)).

In group 2, traditional anterior incision was made just above the frontal and parietal bone, followed by periosteum removal and skin interrupted sutures with 3-0 nonabsorbable sutures (MERSILK ETHICON, Johnson & Johnson Medical. Ltd.). Before knotting, the anterior and posterior terminal points of incision were held by microforceps for exposure of the injection area. Then, 100 *μ*L of UHMWPE particles suspension was injected directly onto the bone using microsyringe, after which the incision was carefully closed interruptedly (see Figures 2(f) to 2(j)). Animals were returned to their cages with water and food ad libitum until euthanasia.

Intraoperative fluid leakage was recorded on the operative day (day 0). Postoperative wound complications were recorded at day 7, including crust formation, wound dehiscence, and bone exposure.

### 2.4. Sampling

The animals were euthanatized by overdose pentobarbital sodium at the end of the study. The calvaria bones were dissected and fixed in 10% buffered formalin. For each group, five samples were decalcified in 10% ethylene diamine tetra acetic acid (EDTA) and embedded in paraffin. The calvaria bones were then cut into 5 *μ*m midfrontal tissue sections, with the midline seams at the center of the cross section. For qualitative histological analysis, the sections were stained with hematoxylin and eosin (H&E).

The other five samples were only fixed in 10% buffered formalin for quantitative evaluation of osteolysis by using micro-CT. The imaging was performed in (Shanghai Showbio Biotech, Inc.). A micro-CT scan was performed ex vivo for 5 animals per group in order to detect changes in bone volume (BV) relative to the total volume (TV), defined as the bone volume fraction (BVF), and measurement of bone mineral density (BMD). Calvaria bones were placed in ventral position in the micro-CT scanner (SkyScan1076) with 18 *μ*m resolution. After scanning, CTAn Analyzer software (Version:1.8.1.4) was used for acquisition and reconstruction of images.

### 2.5. Statistical Analysis

Statistical analyses were performed with the SPSS statistical package, version 13.0 (SPSS Inc., Chicago, IL). Complications rates differences between groups were performed using chi square test. *P* < 0.05 was considered as significant difference. For micro-CT data, we used independent-samples *t*-test to compare groups. A level of significance was set at *P* < 0.05.

## 3. Result

Intraoperative fluid leakage was significantly decreased in group 1 (*P* < 0.05). Only 3 mice in group 1 (paraclip technique) were observed with leakage. Postoperative rate of crust formation in group 1 was 20%, significantly decreased compared with 55% in group 2 (transsuture technique) (*P* < 0.05). No wound dehiscence was observed in group 1, in contrast with 45% in group 2 (*P* < 0.05). The incidence of bone exposure in group 1 was also significantly lower than that of group 2, which has a 30% rate of bone exposure (*P* < 0.05). See Table 1.

**Figure 3:**
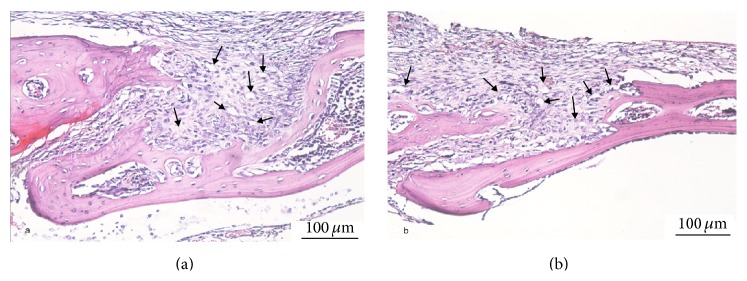
Histological appearance of murine calvaria tissue sections (hematoxylin and eosin staining; magnification: ×200). Inflammatory reactions and osteolysis were demonstrated in group 1 (paraclip) (a) and more more obvious osteolysis in group 2 (transsuture) (b) (black arrows indicate cellular infiltration on bone tissue and around polyethylene particles).

H&E staining demonstrated obvious osteolysis and cellular infiltration of giant cells on bone tissue and around polyethylene particles in group 1 as presented in Figure 3(a) and more obvious osteolysis and increased cellular infiltration of giant cells on bone tissue and around polyethylene particles in group 2 as shown in Figure 3(b).

**Figure 4:**
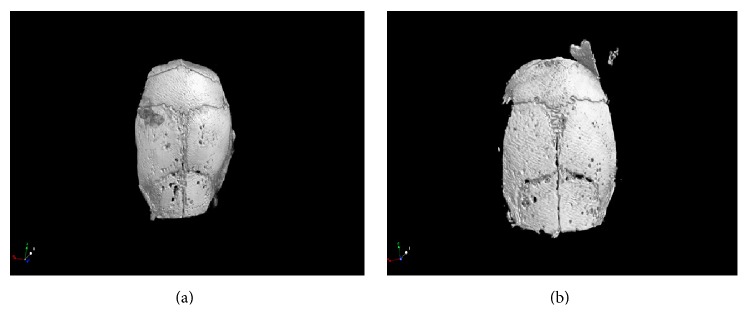
3D microcomputed tomography appearance of murine calvaria osteolysis induced by polyethylene particles by paraclip technique (a) and trans-suture technique (b).

Micro-CT analysis shows osteolytic lesions within the two groups (Figures 4(a) and 4(b)). Using the paraclip technique, we found that the BVF is (0.32 ± 0.03) in group 1 compared to group 2 (transsuture technique) (0.24 ± 0.05), *P* < 0.05. Bone mineral density (BMD) was 1.11 ± 0.03 in group 1 (paraclip technique) compared to group 2 (transsuture technique) (1.01 ± 0.02), *P* < 0.05.

## 4. Discussion

The murine calvaria model was initially described by Merkel et al. and has been widely adopted as a reliable model for investigating osteolysis [10, 11]. However, conventional modeling techniques (transsuture) suffered from severe drawbacks and complications, such as fluid leakage, skin or soft tissue irritation, wound nonhealing, and bone exposure. As skin and soft tissue disorders can directly influence the underlying bone, leading to inflammation and infection, it takes risk to draw conclusion from research results using the traditional surgical techniques. To reduce the complications rates, Rao et al. introduced a percutaneous close injection technique [12]; in their study micro-CT revealed a significant difference in polyethylene group but not in saline group. However, their method does not allow periosteum removal before injection and also does not permit other administration routes of wear particles other than syringe injections. Our micro-CT result indicates that both techniques induce osteolysis, but more obvious in transsuture group.

The aim of this study was to introduce surgical modifications (paraclip technique) of the established model with lower complication rates, which also allow periosteum removal and various administration ways.

Few studies have documented the influence of incision complications like fluid leakage. In our study, paraclip group has lower incidence of fluid leakage (15%), and these leakages were only observed at the syringe inserting point, not from the skin incision, while in the transsuture group fluid leakage occurred in all animals directly from the incision site, which showed that skin clip could provide a satisfying closure. In addition, sutures together with particles caused foreign body reactions in surrounding soft tissue and induced the animals to itch wound for removal. In contrast, the clip can elevate the incision edges away from contacting particles, thus avoiding high rate of wound dehiscence in transsuture group (45%). Another improvement of our modified model is the selection of the incision site which was between ears and without direct contact to the injected area on the calvaria bone. There were also more postoperative bone exposures in the transsuture group due to wound dehiscence, and only 3 mice had wound dehiscence without bone exposure, due to crust formation. In contrast, the results of paraclip group were encouraging, for successfully decreasing the above complication rates.

The authors prefer the modification technique to injection technique also for other reasons. Firstly, several studies have been performed with removal of periosteum [17, 18]. In the current research, the authors support its removal to simulate the environment of joint replacement (intramedullary fixation), and it does not affect the blood supply due to existence of endosteum. Secondly, some researchers in previous studies administered dry particles [10, 19–21], but the close injection method is unsuitable for periosteum removal or dry type particles administration which could only be performed through open surgeries [12]. Modified paraincision injection can provide an appropriate approach for repeated injections, simulating continuous particles generation around artificial joint prosthesis.

There were limitations in this technique, such as the use of flat bone but not long bone and the injection of particles on cortical bone rather than cancellous bone. Another limitation of this technique is the need for two surgeons to complete the procedure. In addition, the model provides acute inflammation rather than the chronic inflammation observed in aseptic loosening in clinical settings. There were also limitations in this study, such as the absence of macrophages quantitative analyzing by immunostaining of CD68 and lack of quantify evaluation for inflammatory reactions, which should be achieved in future studies. The lack of direct quantitative measurement method of fluid leakage is also one of the limitations in this study. The authors concentrated on decreasing incisional complications in the conventional model, and our results showed that the modified technique successfully decreased related peripheral soft tissue inflammatory reactions, which caused more obvious osteolysis reactions in conventional technique group.

## 5. Conclusions

This study indicated that the paraclip modification technique is effective in decreasing complications rates of the conventional murine calvaria osteolysis model. By combining the advantage of posterior incision with clip skin closure and paraincision injections; this modified model provided a simple and reliable method for in vivo evaluation of osteolysis caused by wear debris and related therapeutic studies.

## Supplementary Material

Figure in page 2: The figures show the incision between ears closed by skin clip.
Figure in page 3: The figure shows the normal incision healing absent of inflammation, swelling or other complications.
Figure in page 4: The figures show wound dehiscence and inflamed incision. Bone inflammation & infection secondary to peripheral soft tissue disorders.
Figure in page 5: The schematic illustration indicates the advantages and disadvantages of Conventional surgical technique.
Figure in page 6: The schematic illustration indicates the advantages and disadvantages of Percutaneous injection technique.
Figure in page 7: The schematic illustration indicates the advantages of Modified paraclip technique.

## Figures and Tables

**Table 1:** The statistical analysis of wound complications.

Complications	Paraclip	Transsuture	*χ* ^2^	*P* value
	(*n* = 20)	(*n* = 20)		
Fluid leakage^*∗*^	3 (15.0%)	20 (100.0%)	29.565	0.000
Crust formation^*∗∗*^	4 (20.0%)	11 (55.0%)	5.227	0.022
Wound dehiscence^*∗∗*^	0 (0.0%)	9 (45.0%)	11.613	0.001
Bone exposure^*∗∗*^	0 (0.0%)	6 (30.0%)	7.059	0.008

^*∗*^Day 0.

^*∗∗*^Day 7.

## References

[1] Stea S., Visentin M., Granchi D. (1999). Wear debris and cytokine production in the interface membrane of loosened prostheses. *Journal of Biomaterials Science: Polymer Edition*.

[2] Ingham E., Fisher J. (2000). Biological reactions to wear debris in total joint replacement. *Proceedings of the Institution of Mechanical Engineers, Part H*.

[3] Berry D. J., Harmsen W. S., Cabanela M. E., Morrey B. F. (2002). Twenty-five-year survivorship of two thousand consecutive primary Charnley total hip replacements: factors affecting survivorship of acetabular and femoral components. *The Journal of Bone and Joint Surgery—American Volume*.

[4] Keener J. D., Callaghan J. J., Goetz D. D., Pederson D. R., Sullivan P. M., Johnston R. C. (2003). Twenty-five-year results after Charnley total hip arthroplasty in patients less than fifty years old: a concise follow-up of a previous report. *The Journal of Bone & Joint Surgery—American Volume*.

[5] Nizegorodcew T., Gasparini G., Maccauro G., Todesca A., De Santis E. (1997). Massive osteolysis induced by high molecular weight polyethylene wear debris. *International Orthopaedics*.

[6] Bauer T. W. (2002). Particles and periimplant bone resorption. *Clinical Orthopaedics and Related Research*.

[7] Purdue P. E., Koulouvaris P., Nestor B. J., Sculco T. P. (2006). The central role of wear debris in periprosthetic osteolysis. *HSS Journal*.

[8] Gallo J., Havranek V., Zapletalova J. (2010). Risk factors for accelerated polyethylene wear and osteolysis in ABG I total hip arthroplasty. *International Orthopaedics*.

[9] Langlois J., Hamadouche M. (2011). New animal models of wear-particle osteolysis. *International Orthopaedics*.

[10] Merkel K. D., Erdmann J. M., McHugh K. P., Abu-Amer Y., Ross F. P., Teitelbaum S. L. (1999). Tumor necrosis factor-*α* mediates orthopedic implant osteolysis. *The American Journal of Pathology*.

[11] Kwon Y., Maloney W., Bataractcha T. A simple animal model for particulate debris-induced osteolysis using mouse calvarium.

[12] Rao A. J., Zwingenberger S., Valladares R. (2013). Direct subcutaneous injection of polyethylene particles over the murine calvaria results in dramatic osteolysis. *International Orthopaedics*.

[13] Wooley P. H., Morren R., Andary J. (2002). Inflammatory responses to orthopaedic biomaterials in the murine air pouch. *Biomaterials*.

[14] Fulin P., Pokorny D., Slouf M., Vackova T., Dybal J., Sosna A. (2014). Effect of sterilisation with formaldehyde, gamma irradiation and ethylene oxide on the properties of polyethylene joint replacement components. *Acta Chirurgiae Orthopaedicae et Traumatologiae*.

[15] MacDonald D., Hanzlik J., Sharkey P., Parvizi J., Kurtz S. M. (2012). In vivo oxidation and surface damage in retrieved ethylene oxide-sterilized total knee arthroplasties. *Clinical Orthopaedics and Related Research*.

[16] Costa L., Luda M. P., Trossarelli L., Brach Del Prever E. M., Crova M., Gallinaro P. (1998). Oxidation in orthopaedic UHMWPE sterilized by gamma-radiation and ethylene oxide. *Biomaterials*.

[17] Taki N., Tatro J. M., Lowe R., Goldberg V. M., Greenfield E. M. (2007). Comparison of the roles of IL-1, IL-6, and TNF*α* in cell culture and murine models of aseptic loosening. *Bone*.

[18] Takahashi K., Onodera S., Tohyama H., Kwon H. J., Honma K.-I., Yasuda K. (2011). In vivo imaging of particle-induced inflammation and osteolysis in the calvariae of NFkappaB/luciferase transgenic mice. *Journal of Biomedicine and Biotechnology*.

[19] von Knoch M., Jewison D. E., Sibonga J. D. (2004). Decrease in particle-induced osteolysis in obese (ob/ob) mice. *Biomaterials*.

[20] Nich C., Langlois J., Marchadier A. (2011). Oestrogen deficiency modulates particle-induced osteolysis. *Arthritis Research and Therapy*.

[21] Carmody E. E., Schwarz E. M., Puzas J. E., Rosier R. N., O'Keefe R. J. (2002). Viral interleukin-10 gene inhibition of inflammation, osteoclastogenesis, and bone resorption in response to titanium particles. *Arthritis and Rheumatism*.

