# Supplementary material for: Surgical Modification of the Murine Calvaria Osteolysis Model
Source: Biomed Res Int. 2015 Dec 3;2015:802697. doi: 10.1155/2015/802697 (PMC4681799; doi:10.1155/2015/802697)
Supplement: Supplementary file 1 — Figure in page 2: The figures show the incision between ears closed by skin clip. Figure in page 3: The figure shows the normal incision healing absent of inflammation, swelling or other complications. Figure in page 4: The figures show wound dehiscence and inflamed incision. Bone inflammation & infection secondary to peripheral soft tissue disorders. Figure in page 5: The schematic illustration indicates the advantages and disadvantages of Conventional surgical technique. Figure in page 6: The schematic illustration indicates the advantages and disadvantages of Percutaneous injection technique. Figure in page 7: The schematic illustration indicates the advantages of Modified paraclip technique. [file 802697.f1.pdf]

Surgical modification of the murine calvaria osteolysis model

Supplementary Materials

Ali Mohammed AL-Quhali ,Yu Sun, Xizhuang Bai et al.

Wound closure with skin clip in Group 1 (modified paraclip technique)

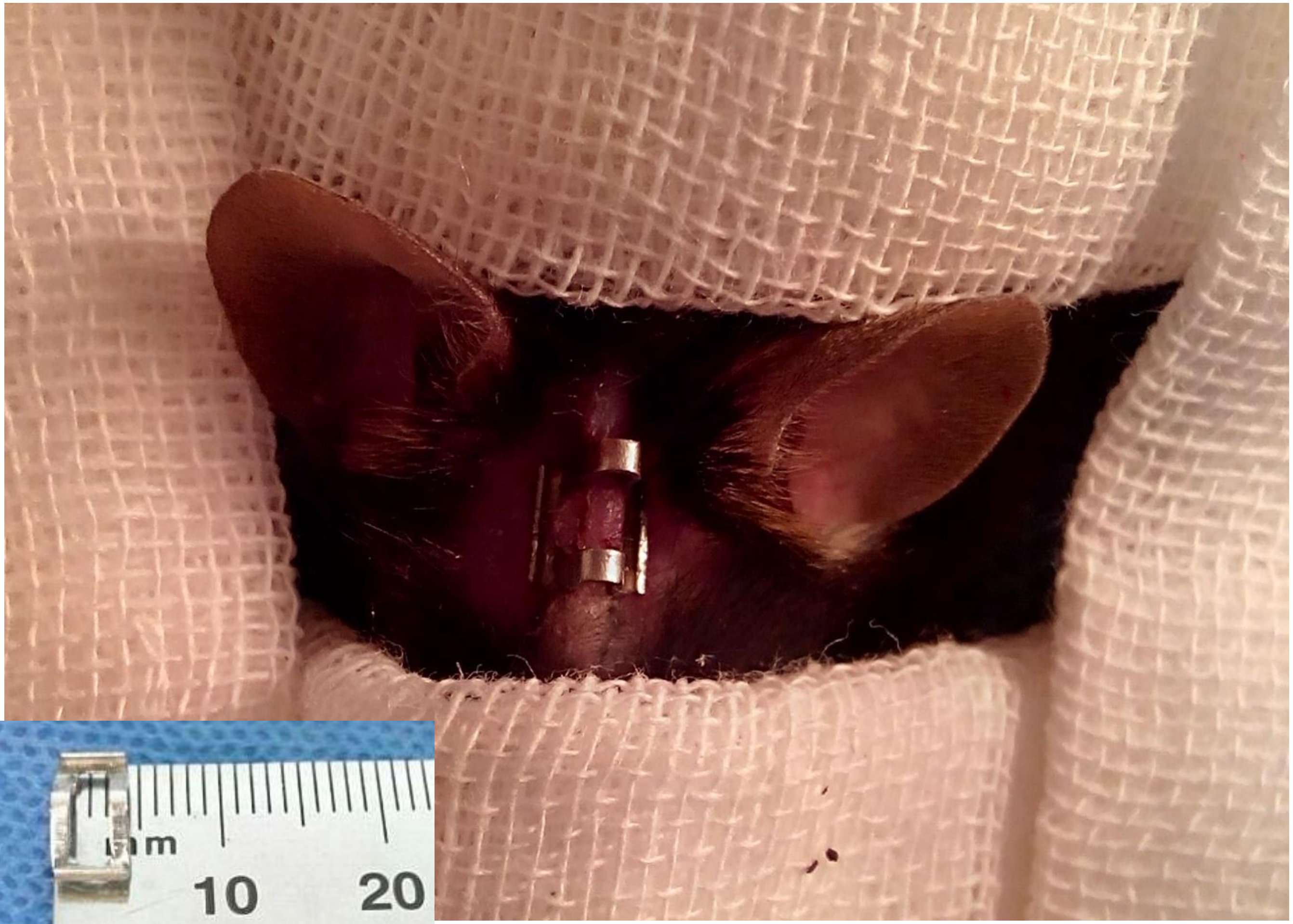

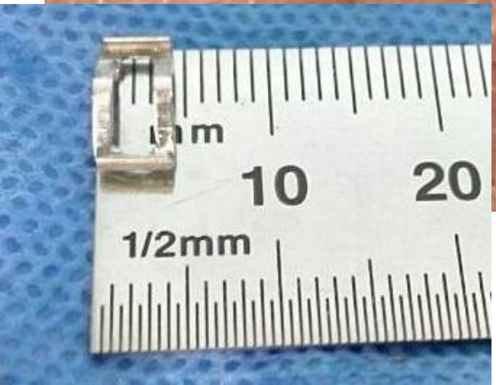

The figures show the incision between ears closed by skin clip.

Normal incision healing after clip removal in Group 1 (modified paraclip technique)

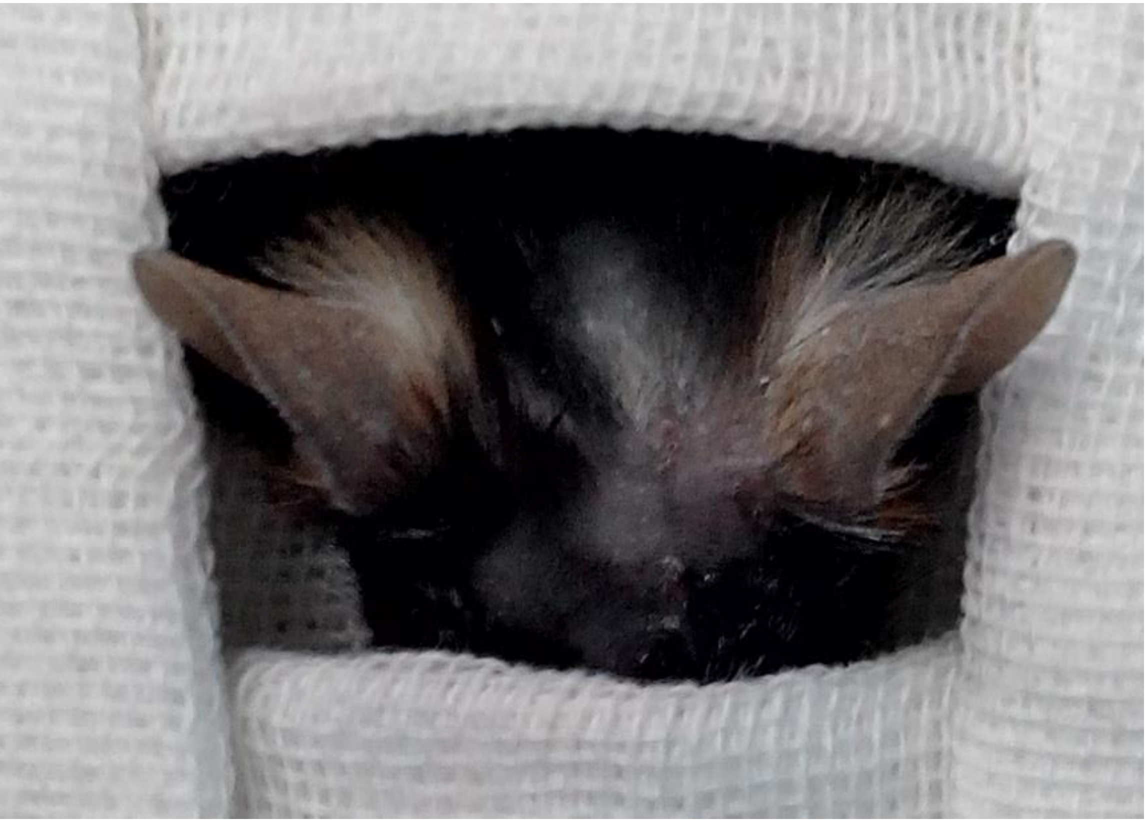

The figure shows the normal incision healing absent of inflammation, swelling or other complications.

Wound dehiscence and bone exposure in Group 2 (conventional transsuture technique)

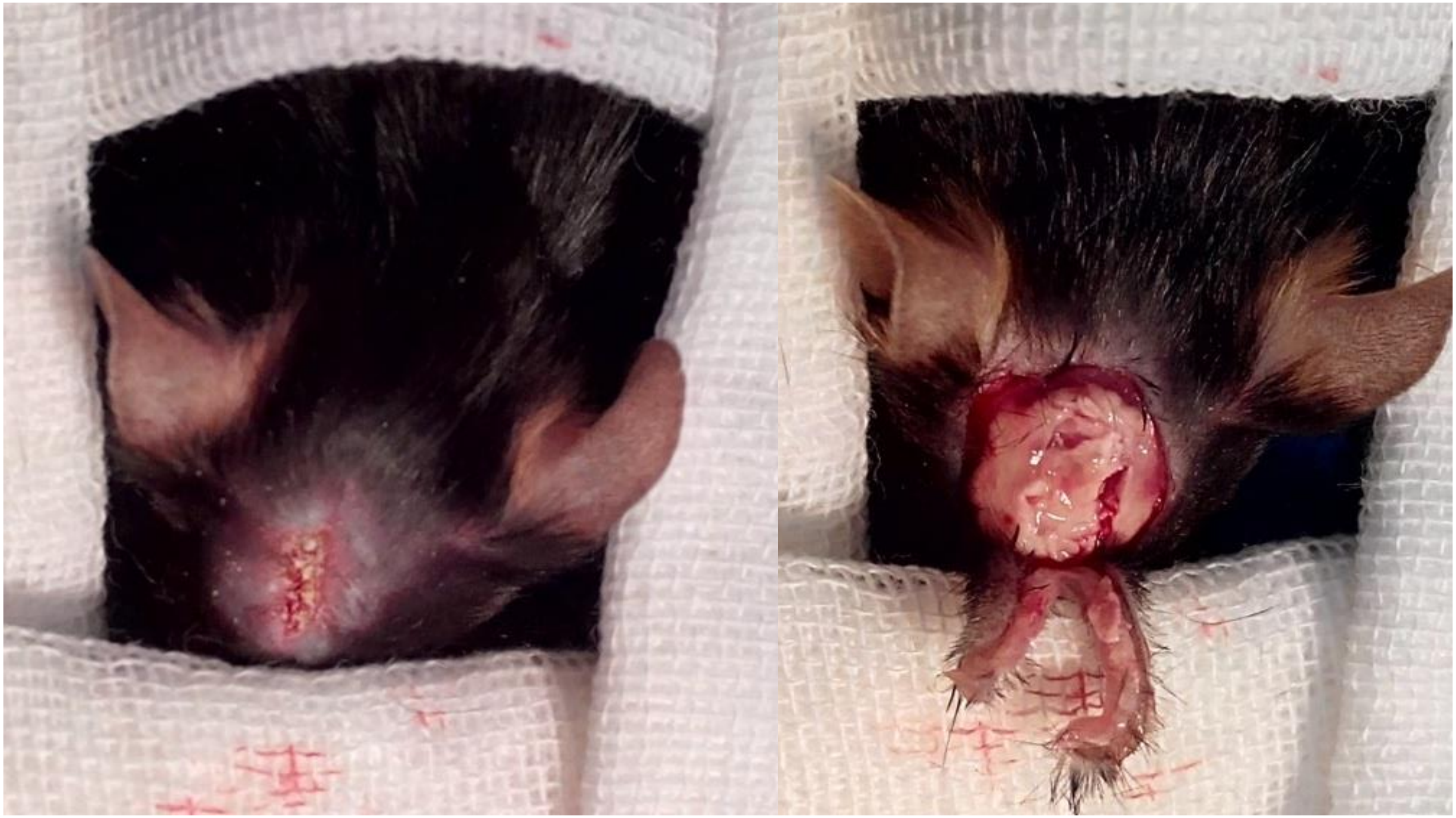

The figures show wound dehiscence and inflamed incision. Bone inflammation & infection secondary to peripheral soft tissue disorders.

Conventional surgical technique

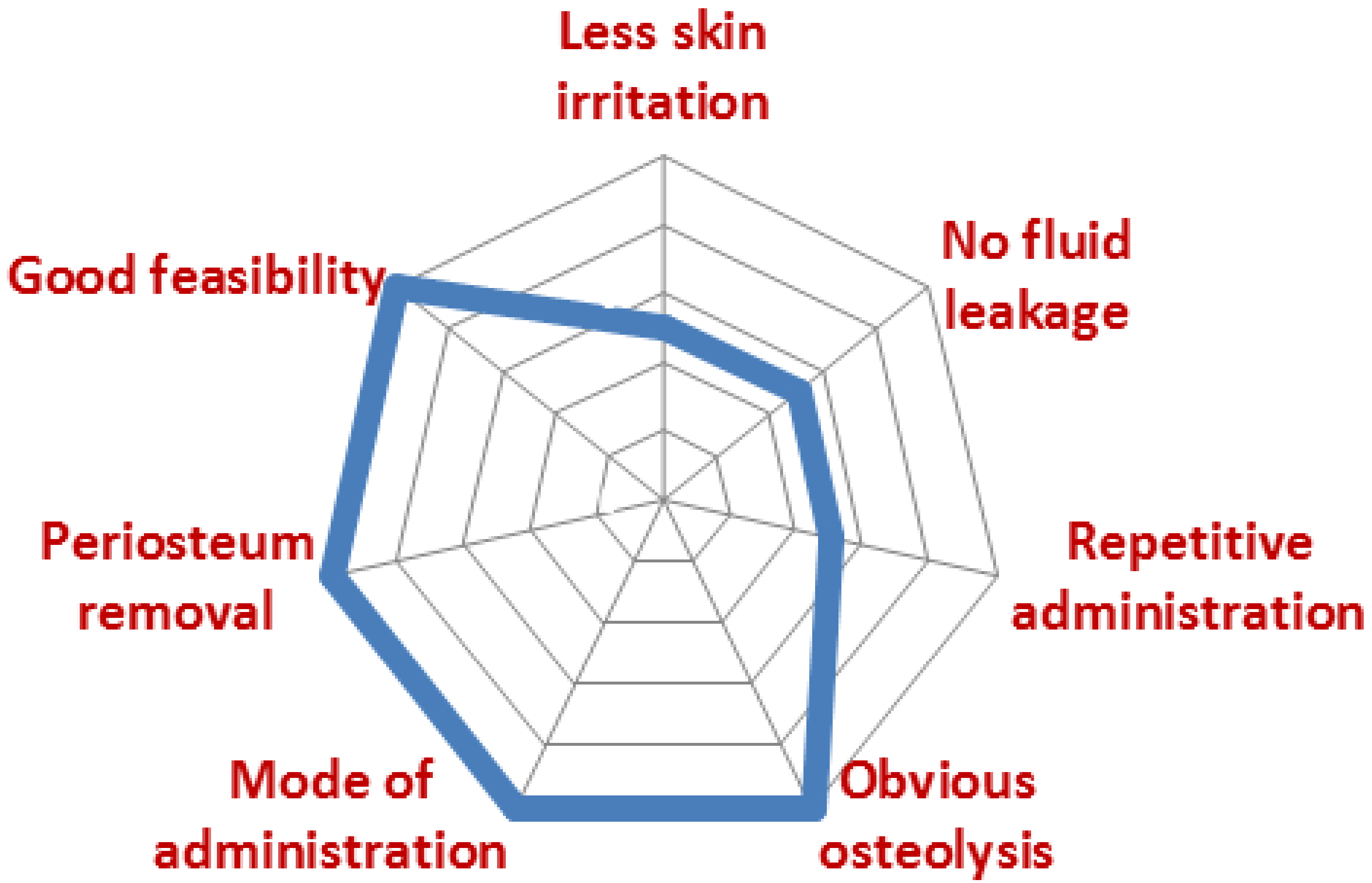

The schematic illustration indicates the advantages and disadvantages of Conventional surgical technique.

Percutaneous injection technique

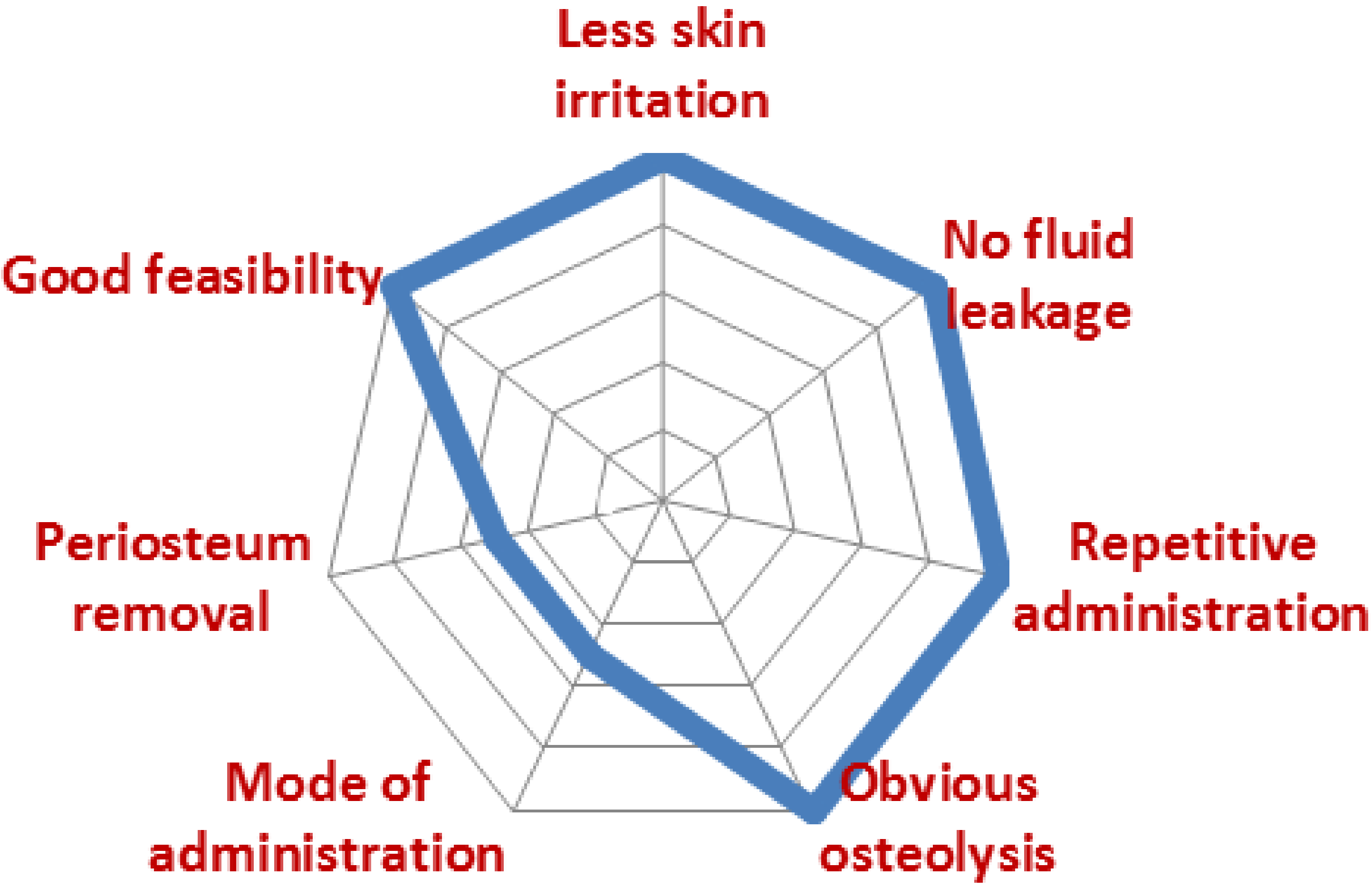

The schematic illustration indicates the advantages and disadvantages of Percutaneous injection technique.

Modified para-clip technique

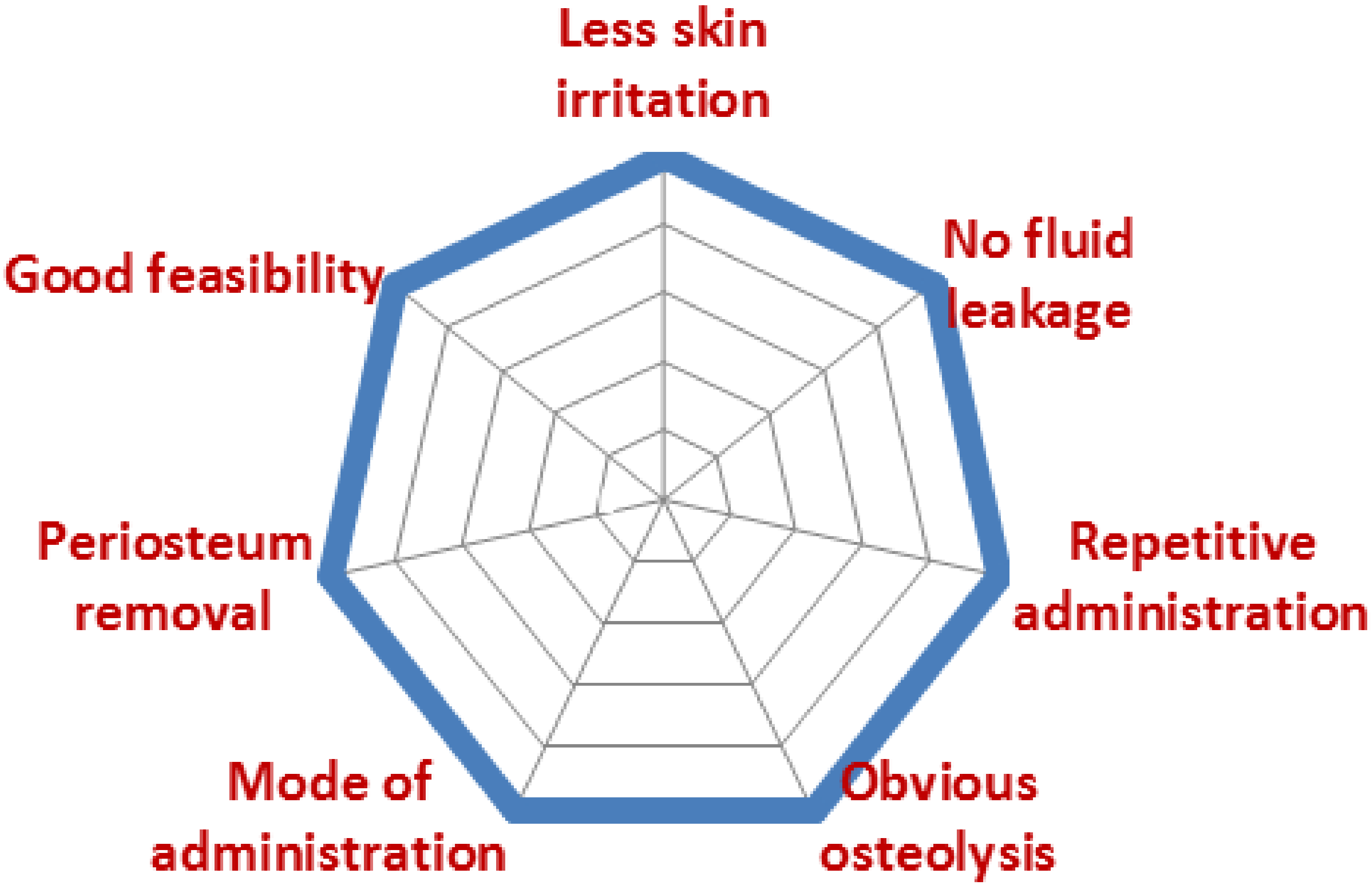

The schematic illustration indicates the advantages of Modified paraclip technique .
